# Incidence and clinical forms of tuberculosis in HIV infected adults: 2013 versus 2007

**DOI:** 10.1186/1471-2334-14-S4-P23

**Published:** 2014-05-29

**Authors:** Olivia Burcoṣ, Simona Erscoiu, Ionuț Cristian Popa, Cristina Pătru, Tatiana Stoicev, Olimpia Nicolaescu, Maria Nica, Andreea Todiran, Grațiela Ţârdei, Emanoil Ceauşu

**Affiliations:** 1 Clinical Hospital of Infectious and Tropical Diseases “Dr. Victor Babeş”, Bucharest, Romania; 2 Carol Davila University of Medicine and Pharmacy, Bucharest, Romania

## 

Tuberculosis (TB), yet an endemic disease in Romania with a high prevalence compared to the average European incidence, had a slight descendent curve. But in the last 3 years we noticed a new phenomenon: an epidemic of tuberculosis among newly HIV infected intravenous drug users (IVDU). Objective: to determine the incidence, clinical and epidemiological characteristics of the new TB cases and TB reactivation in two different years, 2007 and 2013 (before and after HIV epidemic in IVDU) in our clinic of HIV infected adults.

We found a higher incidence of new infected TB cases or TB reactivation in 2013 (45 cases) versus 22 cases in 2007. The average age of patients was lower (33.6 years) in 2013 than in 2007 (35.6). Predominance of male patients was higher in 2013 (79%) vs. 76% in 2007. HIV transmission in 2013: 61% IVDU, 35% sexual transmission, 2% horizontal transmission cohort; in 2007 74% sexually transmitted infection, 14% horizontal infections. In 2013, 41% of HIV-TB coinfected patients were romani ethnics (81% of them IVDU) versus 5% in 2007.

The localization of TB infection was comparable in 2013/2007: disseminated TB 46% vs. 51%, pulmonary TB 51% vs. 50%, peripheral adenopathy 1 case each year.

In 2007 we had 47% patients with sensibility to the 2 major first class drugs – hydrazine (H) and rifampin (R), resistance to H 6%, to R 12%, MDR 6%. In 2013 we have 85% patients HR-sensitive, 3% H-resistant, 3% R-resistant and 10% MDR.

The global mortality of the two groups of patients was higher in 2007 than in 2013 (32% vs. 23%). Deaths related to TB infection were higher in 2013 than in 2007 (89% vs. 57%). Many cases of TB in IVDU HIV infected patients were severe: (58% disseminated TB). Mortality among HIV-TB coinfected IVDU was 50%, versus 8% in non-IVDU patients.

TB had a high prevalence in HIV patients, in 2013 as in 2007, involving significant mortality and morbidity. A lot of newly HIV infected persons are still late and very late presenters. We are expecting that the epidemic of ethnobotanic drugs will raise the prevalence of TB. New techniques of TB antibiogram can reduce mortality.

